# Determinants of condom use among parous women in North Central and South Western Nigeria: a cross-sectional survey

**DOI:** 10.1186/s13104-018-3573-5

**Published:** 2018-07-13

**Authors:** Anthony I. Ajayi, Wilson Akpan

**Affiliations:** Department of Sociology, University of Fort Hare, 50 Church Street, East London, 5200 South Africa

**Keywords:** Condom, Family planning, Contraceptives, South Western Nigeria and North Central Nigeria

## Abstract

**Objectives:**

There appears to be an increasing trend of condom use for pregnancy prevention among nulliparous and multiparous women in developing countries. Drawing from a cross-sectional survey involving 1227 women selected using a 3-stage cluster random sampling technique, the study examines the rates of condom use and its determinants among parous women in three states in North Central and South Western Nigeria.

**Results:**

The rate of condom use among parous women was 13.8% and 23.2% among women using any form of contraceptives. After adjusting for confounding factors (religion and marital status, socioeconomic status and access to a health facility in the resident community), women aged 26–35 (AOR 2.7; CI 1.6–4.5), urban residence (AOR: 3.6; CI 2.2–5.8), no income (AOR: 2.7; CI 1.4–4.9), living in Ekiti State (AOR: 1.8; CI 1.2–2.8) and having a tertiary level of education (AOR: 4.5; CI 1.3–15.6) were the independent predictors of condom use. There is an increasing trend of condom use among parous women.

**Electronic supplementary material:**

The online version of this article (10.1186/s13104-018-3573-5) contains supplementary material, which is available to authorized users.

## Introduction

Condom use has numerous health benefits. Studies have shown that correct and consistent use of condoms is effective in preventing unplanned pregnancy and sexually transmitted infections (STIs) [1, 2]. Of all contraceptive methods, a condom is the only method that protects an individual from acquiring STIs. Condoms have the advantages of ease of access, ease of use, and relatively few side effects compared to other modern contraceptive methods.

However, several barriers impinge on the use of condoms, among which are the perceived reduction in sexual pleasure, a negative cultural norm about condoms, religion, gender inequality, lack of partner communication, lack of motivation, cost and lack of knowledge [3–9]. A Canadian study showed that people were more likely to rate their most recent sex as “very pleasurable” when condoms were not used compared to when condoms were used [10]. There is also evidence that the condom has negative symbolism in some sub-Saharan Africa settings [8]. In these settings, some people believe that condom use promotes sexual promiscuity [11], or is suggestive of filth, disease, infidelity and mistrust [8]. Sarkar claims that the stigma attached to the use of condoms in these settings could prevent women from using condoms [3].

Studies have shown that condom use self-efficacy, autonomy, partner communication, positive attitude to condoms and condom use skills promote condom use [12–16]. Condom use varies by age, sex, race, marital status, level of education, place of residence, relationship types and number of sexual partners [17–20]. A national survey in the United States showed that only 23.8 and 33.7% of women and men aged 15–44 used a condom at their last sexual intercourse [17]. However, higher condom use prevalence was reported among unmarried adults compared to married adults, among adolescents compared to adults, people living with HIV and commercial sex workers [21–23]. This is unsurprising considering that almost all interventions to promote condom use are targeted at high-risk populations [24]. Women who reside in urban areas are more likely to use condoms compared to those living in rural areas [19]. Urban women tend to display a positive attitude toward condoms and demonstrate high self-efficacy to condom use [19]. Being female, having a regular partner, low socioeconomic status, high coital frequency, alcohol use, and dating an older partner are factors associated with low or inconsistent condom use [25, 26].

There is evidence of an increasing trend of condom use for pregnancy prevention among women in sub-Saharan Africa [27]. Even though the Nigerian Demographic and Health Survey reports a low rate of condom use (2.1% among currently married women and 40% among sexually active unmarried women), many studies have shown that a condom is the most used contraceptive method in Nigeria [28–31]. However, factors influencing the use of condoms among childbearing women in Nigeria, especially in the South Western and North Central regions, are less understood. Besides, most studies on condom use have focused on high-risk populations such as men having sex with men, commercial sex workers and adolescents. It is against this background that this study aims to establish the rates of condom use and its determinants among childbearing women in a North Central and two South Western Nigerian states.

## Main text

### Methods

#### Study area

The data analysed in this paper was derived from a larger study, which investigated maternal outcomes in the context of free maternal health care (MANCONFREE Study) in Ondo and Ekiti States in South Western Nigeria and in the Nasarawa State in North Central Nigeria. The study took place between May and September 2016. The populations of Ekiti and Ondo States in 2011, according to the National Bureau of Statistics (NBS), were 2,801,887 and 4,020,965 respectively. Nasarawa State is one of the six states in North Central Nigeria. According to the NBS, the population of Nasarawa State was 2,171,908 people in 2011. These three states were selected due to their unique maternal health care policies and varying maternal health outcomes.

#### Study participants and inclusion criteria

The population of interest in this study was women within the reproductive age (age 18–49 years). The inclusion criterion was women who gave birth to at least one child between the years 2011 and 2015. Women within the reproductive age, who had never given birth to any children or who did not give birth during the period in review, were excluded. Nulliparous women were excluded in the bigger study, which evaluated free maternal health care policy in North Central and South Western Nigeria, because only parous women views and experiences are relevant to evaluating maternal outcomes in the context of free maternal health care.

#### Study design and sample selection

This was a descriptive, cross-sectional study. Questionnaires were administered to 1227 women using the face-to-face interview method [32]. The full details of the methodology and sampling design can be found elsewhere [33]. The sample size calculator [34] was used to estimate the appropriate sample size for the study. A sample size of 409 was estimated per state, adjusted for missing responses and at a confidence level of 95%, confidence interval of ± 5, and using an infinite population. Infinite population was used because the total number of women who gave birth over the 5-year period could not be determined. A three-stage cluster random sampling method was used to select a representative sample of women in each of the three states included in this study. Study areas were clustered into enumeration areas (EAs) and stratified into rural, peri-urban and urban areas. The study took place in a total of 81 EAs. The list of EAs in the 2006 census was used in this study. However, every 10th household in each enumeration area was visited to identify study participants until the sample size of 1227 women was reached. This enabled the researchers to include new houses found in the EAs. Households without women who gave birth during the specified period were skipped; and only one woman was selected in a household, irrespective of the number of “eligible” women there.

#### Method of data collection

The questionnaire was piloted among 20 women who were included in the main study. Questions included in the questionnaire probed participants’ demographic characteristics, use of maternal health care services, perception of maternal health care services, users’ experience, level of satisfaction with services, knowledge of contraceptives methods, use of contraceptive methods and contraceptive discontinuation (see Additional file 1).

#### Measurements

Condom use was operationalised by asking what method participants used to prevent pregnancy at the time they did not want to become pregnant. Responses were recoded to condom users and non-condom users. Other variables of interest in this article included age, marital status, income, level of education, place of residence, religion, regularity of viewing television, access to Internet and ownership of mobile phone and bank accounts.

#### Method of data analysis

Data were analysed using the Statistical Package for Social Sciences, version 24. Simple frequencies of all variables of interests were computed and tabulated. The mean age and standard deviation were calculated. To examine demographic correlates of condom use, Pearson Chi square and binary logistic regression were employed. All significant variables at alpha value less than 0.05 in the Pearson Chi square statistics were included in the binary logistic regression analysis. Binary logistic regression analysis was performed at a confidence level of 95%.

### Results

The average age of participants was 30.8 years (SD ± 6.3). Most participants were married (95.9%), Christians (76.9%), owned a mobile phone (89.1%), and watched television regularly (91.6%). Few participants were above 40 years (5.7%), never married (3.1%), and had no formal education (7.7%) (Table 1).

**Table 1:** Demographic characteristic of study participants

Variables	Frequency	Percent
State		
Ekiti	400	33.0
Ondo	402	33.1
Nasarawa	411	33.9
Age groups		
20 and below	69	5.7
21–25	239	19.8
26–30	368	30.4
31–35	276	22.8
36–40	189	15.6
40 and above	69	5.7
Marital status		
Currently married	1163	5.9
Formerly married	12	1.0
Never married	38	3.1
Residence		
Urban	384	31.7
Peri-urban	339	27.2
Rural area	499	41.1
Religion		
Christianity	933	76.9
Islam	276	22.8
Traditional	4	0.3
Level of education		
No formal education	93	7.7
Primary education	207	17.1
Secondary education	572	47.2
Tertiary education	339	28.0
Levels of income		
No income	338	28.5
Below 20,000 Naira	693	58.4
Above 20,000 Naira	156	13.1
Own a mobile phone		
Yes	1081	89.1
No	132	10.9
Watch television regularly		
Yes	1111	91.6
No	102	8.4
Own a bank account		
Yes	602	49.6
No	611	50.4
Use the internet		
Yes	338	27.9
No	875	72.1
Socioeconomic status		
Low	201	16.9
Middle	611	51.5
High	374	31.5

#### Condom use and associated factors among childbearing women

The prevalence of condom use among childbearing women was 13.8% (Additional file 2: Figure S1). About one in five women who were contraceptive users reported the use of a condom. Condom use was significantly associated with age, place of residence, level of education, level of income, availability of a health facility in the resident community and socioeconomic status (Table 2).

**Table 2:** Demographic determinants of condom use among childbearing women in Southwestern and North Central Nigeria

Variables	All women n = 1213		*p* value
	Yes n (%)	No n (%)	
State			
Ekiti	65 (16.3)	335 (83.8)	0.013
Ondo	39 (9.7)	363 (90.3)	
Nasarawa	64 (15.6)	347 (84.4)	
Age groups			
20 and below	5 (7.2)	64 (92.8)	0.002
21–25	31 (13.0)	208 (87.0)	
26–30	68 (18.3)	303 (81.7)	
31–35	45 (16.3)	231 (83.7)	
36–40	14 (7.4)	175 (92.6)	
40 and above	5 (7.2)	64 (92.8)	
Marital status			
Currently married	159 (13.7)	1004 (86.3)	0.678
Formerly married	2 (16.7)	10 (83.3)	
Never married	7 (18.4)	31 (81.6)	
Residence			
Urban	72 (18.8)	312 (81.3)	< 0.001
Peri-urban	65 (19.6)	266 (80.4)	
Rural area	31 (6.2)	467 (93.8)	
Religion			
Christianity	140 (15.0)	793 (85.0)	0.088
Islam	28 (10.1)	248 (89.9)	
Traditional	0 (0.0)	4 (100.0)	
Level of education			
No formal education	3 (3.2)	90 (96.8)	< 0.001
Primary education	24 (11.6)	183 (88.4)	
Secondary education	76 (13.3)	496 (86.7)	
Tertiary education	65 (19.2)	274 (80.8)	
Levels of income			
No income	67 (19.8)	271 (80.2)	0.001
Below 20,000 Naira	79 (11.4)	614 (88.6)	
Above 20,000 Naira	22 (14.1)	134 (85.9)	
Socioeconomic status			
Low	25 (12.4)	176 (87.6)	0.043
Middle	76 (12.4)	535 (87.6)	
High	67 (17.9)	307 (82.1)	
Availability of health care facility in resident community			
Yes	157 (15.0)	891 (85.0)	0.004
No	11 (6.7)	154 (93.3)	

In the logistic regression, after adjusting for confounding factors (socioeconomic status and access to a health facility in the resident community, religion and marital status), women aged 26–35 (AOR 2.7; CI 1.6–4.5), urban residence (AOR: 3.6; CI 2.2–5.8), no income (AOR: 2.7; CI 1.4–4.9), living in Ekiti State (AOR: 1.8; CI 1.2–2.8) and having a tertiary level of education (AOR: 4.5; CI 1.3–15.6) were the independent predictors of condom use (Table 3).

**Table 3:** Binary logistic regression showing determinants of condom use among childbearing women

Variables	β	Wald	AOR (CI)	p value
Age groups				
25 and below	0.4	1.9	1.6 (0.8–2.9)	0.164
26–35	1.0	13.4	2.7 (1.6–4.5)	0.000
36 and above (reference)				
Level of education				
Tertiary education	1.5	5.5	4.5 (1.3–15.6)	0.019
Secondary education	1.3	4.0	3.5 (1.0–12.0)	0.047
Primary education	1.2	3.7	3.5 (1.0–12.3)	0.054
No formal education (reference)				
Levels of income				
No income	1.0	9.8	2.7 (1.4–4.9)	0.002
Below 20,000	0.2	0.3	1.2 (0.7–2.0)	0.559
Above 20,000 (reference)				
State				
Ekiti state	0.6	6.7	1.8 (1.2–2.8)	0.01
Nasarawa state	0.5	4.2	1.7 (1.0–2.7)	0.04
Ondo state (reference)				
Place of residence				
Urban	1.3	26.5	3.6 (2.2–5.8)	0.000
Peri-urban	1.2	23.4	3.3 (2.0–5.4)	0.000
Rural (reference)				

*AOR* adjusted odd ratio, *CI* confidence interval

### Discussion of findings

This study aimed to establish the rates and determinants of condom use among childbearing women in Nasarawa State in North Central Ondo and the Ekiti States of South Western Nigeria. Overall, only 13.8% women reported current use of condoms. About one out of five women who reported using any contraceptive methods were using condoms. The rate of contraceptive use varied by state with Ondo having the least prevalence of condom use. Younger age, urban residence, no monthly income, Ekiti and Nasarawa States’ residency and having a tertiary education were the independent predictors of condom use. The rates of condom use in each state found in this study were higher than those reported in the 2013 NDHS. Perhaps the use of condoms has increased among childbearing women. A study has reported that there is an increasing trend of condom use for pregnancy prevention among married women in sub-Saharan Africa [27]. Even though the rate of condom use found in our study is still low, our data present evidence of increasing condom use trend for pregnancy prevention among parous women in the study settings. In all of the three states surveyed, condom use increased notably when compared with NDHS 2013. For instance, condom use among parous women in Ekiti State in the year 2013 was 8.2% [35] compared to the 16.3% in the present study.

Our study shows that condom use was higher among young adults compared to older adults, which is consistent with what was previously reported [17–20]. Younger adults could perhaps have favourable attitudes towards the use of condoms. It could also be that younger adults preferred condoms to hormonal contraceptives because they dread their side effects. There is evidence showing that hormonal contraceptive use is lowest among younger adults [36, 37]. A study conducted in the study settings shows that young adults believe that hormonal contraceptives are harmful to the body and could compromise their ability to conceive in the future [36, 37].

Another finding of this study is that urban women were more likely to report current use of contraceptives compared to rural women. This finding is consistent with previous studies [15, 19, 23]. A probable explanation for this could be that urban women have higher condom use self-efficacy and favourable attitudes to condom use. Adherence to cultural norms is generally stronger in rural areas due to the homogenous nature of solidarity among rural residents. Thus, accessing condoms in a rural setting is less confidential compared to accessing them in an urban area. It is also plausible that urban women enjoy relatively better access to condoms than women who reside in rural areas. The finding further illustrates the urban advantage in the access and use of health care services. Efforts to improve contraceptives’ use, especially condom use, should be targeted in rural areas.

One surprising finding of this study is that the women who reported no income were more likely to be current users of condoms compared to women who reported some income. One plausible explanation for this finding could be that women who reported no income are young and highly educated. Inferring from the study data, condom use was higher among younger women and those with tertiary education. It is possible that young, educated women are unemployed, but could still access condoms freely. There are many non-governmental organisations distributing condoms freely in the study settings. Besides, a highly subsidized condom is available in the study settings.

It is important to note that in a patriarchy society like Nigeria, men play a significant role in condom use. The decision on condom use among childbearing women significantly rest on the men. However we did not include men in this study nor examine attitude towards condom use. Future studies should focus on these identified areas.

### Conclusion

Our study shows that condom use was low among childbearing women and that younger age, urban residence, no monthly income and tertiary education were the determinants of condom use in the study settings.

## Limitations

A causal explanation between age, income, education, place of residence, and condom use cannot be established due to the cross-sectional nature of the study. The authors cannot exclude social desirability bias in condom use due to the self-reporting utilised for data collection. Thus, the rate of condom use may have been under-reported among participants. This study did not examine attitude of men and women to condom use, which is very important towards comprehensive understanding of condom use behaviour of childbearing women. Future study should consider exploring whether there is changing attitude towards condom use among men and women within the reproductive age.

## Additional files

**Additional file 1.** Questionnaire.

**Additional file 2: Figure S1.** Condom use prevalence.

## Abbreviations

EA: enumeration areas
NDHS: Nigerian demographic and health surveys
STIs: sexually transmitted infections
