# Supplementary material for: Determinants of condom use among parous women in North Central and South Western Nigeria: a cross-sectional survey
Source: BMC Res Notes. 2018 Jul 13;11:467. doi: 10.1186/s13104-018-3573-5 (PMC6044001; doi:10.1186/s13104-018-3573-5)
Supplement: Supplementary file 1 — Additional file 1. Questionnaire. [file 13104_2018_3573_MOESM1_ESM.docx]

**ETHICS RESEARCH CONFIDENTIALITY AND INFORMED CONSENT FORM**

I Anthony Ajayi of the department of sociology, University of Fort Hare is conducting research regarding **maternal outcomes in the context of free maternal healthcare: perceptions and realities in south western and north central Nigeria.** I am interested in finding out more about the perception, beliefs, and experiences of women of reproductive age on use of free maternal healthcare services.

Please understand that you are not being forced to take part in this study and the choice whether to participate or not is yours alone. However, we would really appreciate it if you do share your thoughts with us. If you choose not take part in answering these questions, you will not be affected in any way. If you agree to participate, you may stop me at any time and tell me that you don’t want to go on with the interview. If you do this there will also be no penalties and you will NOT be prejudiced in ANY way. Confidentiality will be observed professionally.

I will not be recording your name anywhere on the questionnaire and no one will be able to link you to the answers you give. Only the researchers will have access to the unlinked information. The information will remain confidential and there will be no “come-backs” from the answers you give.

The interview will last around thirty minutes*.* I will be asking you questions and ask that you are as open and honest as possible in answering these questions. Some questions may be of a personal and/or sensitive nature. I will be asking some questions that you may not have thought about before, and which also involve thinking about the past or the future. We know that you cannot be absolutely certain about the answers to these questions but we ask that you try to think about these questions. When it comes to answering questions there are no right and wrong answers. If possible, I would like to come back to this area once we have completed our study to inform you and your community of what the results are and discuss our findings and proposals around the research and what this means for people in this area.

**INFORMED CONSENT by Participant**

I hereby agree to participate in research probing the perception, beliefs about free, use of maternal healthcare services, and experience of mothers using free maternal healthcare. Understand that I am participating freely and without being forced in any way to do so. I also understand that I can stop this interview at any point should I not want to continue and that this decision will not in any way affect me negatively.

I understand that this is a research project whose purpose is not necessarily to benefit me personally.

I have received the telephone number of a person to contact should I need to speak about any issues, which may arise in this interview.

I understand that this consent form will not be linked to the questionnaire, and that my answers will remain confidential.

I understand that if at all possible, feedback will be given to my community on the results of the completed research.

……………………………..

**Signature of participant Date**: …………………

**QUESTIONNAIRE**

**SECTION 1. RESPONDENT'S BACKGROUND**

Please select the appropriate option from the list of options provided

| No | Questions and filters | Coding Categories | Code | Skip |
| --- | --- | --- | --- | --- |
| 01 | Place of residence | City  Town  Rural area | 1  2  3 |  |
| 02 | In what month and year were you born? | Month…………   \|  \|  \| \| --- \| --- \|   Don't know month…………………..  Year   \|  \|  \|  \|  \| \| --- \| --- \| --- \| --- \|   Don't know year ………………….. | 98 |  |
| 03 | How old were you at your last birthday? | Age in completed years   \|  \|  \| \| --- \| --- \| |  |  |
| 04 | Have you ever attended school? | Yes ………………………. …………  No…………………………………… | 1  2 |  |
| 05 | What is the highest level of school you attended: primary, secondary, or higher? | Primary……………………………..  Secondary…………………………..  Higher……………………………….. | 1  2  3 |  |
| 06 | What is your religion | Christianity…………………………  Islam………………………………..  Traditional………………………….  Others Secify………………………………….. | 1  2  3  4 |  |
| 07 | Ethnic Group | Please specify  ………………………… | 96 |  |
| 08 | What is your marital status | Currently married …………………  Formerly married ………...……..  Never been married. ……………… | 1  2  3 |  |
| 09 | Are you employed in a paid job | Yes  No | 1  2 |  |
| 10 | How much is your monthly income | ------------------- |  |  |
| 11 | Do you own a mobile phone | Yes  No | 1  2 |  |
| 12 | Do you watch TV regularly | Yes  No | 1  2 |  |
| 13 | Do you own a bank account | Yes  No | 1  2 |  |
| 14 | Have you ever used the internet | Yes  No | 1  2 |  |
| 15 | How many child/children do you have | \|  \|  \| \| --- \| --- \| |  |  |
| 16 | Number of children between 2011-2015 | \|  \|  \| \| --- \| --- \| |  |  |
| 17 | What year were they born | First   \|  \|  \|  \|  \| \| --- \| --- \| --- \| --- \|   Second   \|  \|  \|  \|  \| \| --- \| --- \| --- \| --- \|   Third   \|  \|  \|  \|  \| \| --- \| --- \| --- \| --- \| |  |  |

**SECTION 2. PREGNANCY AND POSTNATAL CARE ONE OR MORE BIRTHS IN 2010 -2015**

| Now I will like to ask questions about your children born in the last five years (we will talk about each separately) | | | |  |
| --- | --- | --- | --- | --- |
| No |  | Last birth | Next to last birth | Code |
| 18 | Birth History number |  |  |  |
| 19 | When you got pregnant with (name), did you see anyone for antenatal care for this pregnancy? | Yes  No | Yes  No | 1  2 |
| 20 | Whom did you see?  Anyone else?  Probe to identify each type of person and record all mention | **Health personnel**  Doctor  Nurse/Midwife  Auxiliary Midwife  **Other Person**  Traditional birth attendant  Community/village health worker  Others ____________Specify | **Health personnel**  Doctor  Nurse/Midwife  Auxiliary Midwife  **Other Person**  Traditional birth attendant  Community/village health worker  Others____________Specify | **1**  **2**  **3**  **4**  **5**  **96** |
| 21 | Where did you receive antenatal care for this pregnancy?  Anywhere else?  PROBE TO IDENTIFY THE TYPE OF SOURCE. | **Home**  Her Home  Other home  **Public Sector**  Federal Medical Centre  State specialist Hospital  General Hospital  Primary Health Centre  Maternity  **Private medical sector**  Private Hospital/clinic  Other private medical sector  -------------------------  specify  Others ------------------------  Specify | **Home**  Her Home  Other home  **Public Sector**  Federal Medical Centre  State specialist Hospital  General Hospital  Primary Health Centre  Maternity  **Private medical sector**  Private Hospital/clinic  Other private medical sector  -------------------------  specify  Others ------------------------  Specify | 1  2  3  4  5  6  7  8  96  96 |
| 22 | How many months pregnant were you when you first receive antenatal care for this pregnancy? | \|  \|  \| \| --- \| --- \|   Months  Don’t Know ……………. | \|  \|  \| \| --- \| --- \|   Months  Don’t Know ……………. | 98 |
| 23 | How many times did you receive antenatal care during this pregnancy? | \|  \|  \| \| --- \| --- \|   No of times  Don’t Know ……………. | \|  \|  \| \| --- \| --- \|   No of times  Don’t Know ……………. | 98 |
| 24 | As part of your antenatal care during this pregnancy, were any of the following done at least once:   1. Was your blood pressure measure? 2. Did you give a urine sample 3. Did you give a blood sample 4. Were you given injection in the arm to prevent the baby from getting tetanus, that is convulsion after birth | Yes No   1. BP.. 1 2 2. Urine… … 1 2 3. Blood … 1 2      1. Inject ……. 1 2 | Yes No   1. BP.. 1 2 2. Urine… … 1 2 3. Blood … 1 2 4. Inject ……. 1 2 |  |
| 25 | Who assisted with the delivery of (NAME)?  Anyone else?  PROBE FOR THE TYPE(S) OF PERSON(S) AND RECORD ALL MENTIONED.  IF RESPONDENT SAYS NO ONE ASSISTED, PROBE TO DETERMINE WHETHER ANY ADULTS WERE PRESENT AT THE DELIVERY. | **Health personnel**  Doctor  Nurse/Midwife  Auxiliary Midwife  **Other Person**  Traditional birth attendant  Relative/Friend  Others ____________Specify  No one assisted ----------- | **Health personnel**  Doctor  Nurse/Midwife  Auxiliary Midwife  **Other Person**  Traditional birth attendant  Relative/Friend  Others ____________Specify  No one assisted ----------- | 1  2  3  4  5  96  7 |
| 26a | Where did you give birth to (NAME)?  PROBE TO IDENTIFY THE TYPE OF SOURCE.  IF UNABLE TO DETERMINE IF PUBLIC OR PRIVATE SECTOR, WRITE THE NAME OF THE PLACE.  ‑‑‑‑‑‑‑‑‑‑‑‑‑‑‑‑‑‑‑‑‑‑‑‑‑‑‑‑‑‑‑‑‑‑‑‑‑‑‑‑‑‑‑‑‑‑‑  Name of place | **Home**  Her Home  Other home  **Public Sector**  Federal Medical Centre  State specialist Hospital  General Hospital  Primary Health Centre  Maternity  **Private medical sector**  Private Hospital/clinic  Other private medical sector  -------------------------  specify  **Others** ------------------------  Specify | **Home**  Her Home  Other home  **Public Sector**  Federal Medical Centre  State specialist Hospital  General Hospital  Primary Health Centre  Maternity  **Private medical sector**  Private Hospital/clinic  Other private medical sector  -------------------------  specify  **Others** ------------------------  specify | 1  2  3  4  5  6  7  8  9  96 |
| 26b | If at home, probe the choice of home birth by asking why do you give birth at home? | **No money for hospital bill**  **To late to get to hospital**  **Lack of transportation**  **Not necessary**  **Others specify --------------------** | **No money for hospital bill**  **To late to get to hospital**  **Lack of transportation**  **Not necessary**  **Others specify --------------------** | **1**  **2**  **3**  **4**  **96** |
| 27 | How long after (NAME) was delivered did you stay there?  IF LESS THAN ONE DAY, RECORD HOURS; IF LESS THAN ONE WEEK, RECORD DAYS. | Hours ----------  Days -----------  Weeks ----------  Don’t Know | Hours ----------  Days -----------  Weeks ----------  Don’t Know | 1  2  3  98 |
| 28 | Was (NAME) delivered by caesarean, that is, did they cut your belly open to take the baby out? | Yes ……………  NO. .………………. | Yes ……………  NO……………… | 1  2 |
| 29 | I would like to talk to you about checks on your health after delivery, for example, someone asking you questions about your health or examining you. Did anyone check on your health while you were still in the facility? | Yes ………………… No…………………. | Yes ……………….. No…………………  Skip to the next section | 1  2 |
| 30 | How long after delivery did the first check take place?  IF LESS THAN ONE DAY, RECORD HOURS; IF LESS THAN ONE WEEK, RECORD DAYS. | Hours ----------  Days -----------  Weeks ----------  Don’t Know………….. | Hours…………………  Days ………..………  Weeks ………….……..  Don’t Know………….. | 98 |
| 30 | Who checked on your health at that time?  PROBE FOR MOST QUALIFIED PERSON. | **Health personnel**  Doctor  Nurse/Midwife  Auxiliary Midwife  **Other Person**  Traditional birth attendant  Relative/Friend  Others ____________Specify | **Health personnel**  Doctor  Nurse/Midwife  Auxiliary Midwife  **Other Person**  Traditional birth attendant  Relative/Friend  Others ____________Specify | 1  2  3  4  5  96 |

**SECTION 3 PERCEPTIONS OF FREE MATERNAL HEALTHCARE ITEMS**

Please state the extent to which you agree or disagree with the following statements by choosing from Strongly Agree SA, Agree A, Not Sure NS, Disagree D, Strongly Disagree SD.

|  |  | SA | A | NS | D | SD |
| --- | --- | --- | --- | --- | --- | --- |
| 31 | Free maternal healthcare makes the cost of child delivery affordable for me |  |  |  |  |  |
| 32 | I do not have to seek maternal healthcare services in private hospitals because of free maternal healthcare services |  |  |  |  |  |
| 33 | Free maternal health care is easily accessible |  |  |  |  |  |
| 34 | Good quality of services is offered in government hospitals offering free child delivery |  |  |  |  |  |
| 35 | There is no difference in the quality of care in hospitals offering free delivery compared to hospitals that charge fees |  |  |  |  |  |
| 36 | I can trust the quality of care offered under free maternal healthcare programme of the state government |  |  |  |  |  |
|  | **Perception of quality of care under free maternal healthcare**  **Health personnel practices and conduct** |  |  |  |  |  |
| 37 | The waiting time is moderate in government owned hospitals offering free maternal healthcare services |  |  |  |  |  |
| 38 | Doctors are usually friendly in government owned hospitals offering free delivery |  |  |  |  |  |
| 39 | Nurses are usually friendly in government owned hospitals offering free delivery |  |  |  |  |  |
| 40 | The doctors show respect for pregnant women in government owned hospitals offering free maternal healthcare |  |  |  |  |  |
| 41 | The nurses show respect for pregnant women in government owned hospitals offering free maternal healthcare |  |  |  |  |  |
| 42 | The doctor and nurses do a good clinical examination of pregnant women in government owned hospitals offering free maternal healthcare |  |  |  |  |  |
|  | **Adequacy of resources and services** |  |  |  |  |  |
| 43 | Government owned hospitals offering free maternal healthcare have all the medicines needed by pregnant women |  |  |  |  |  |
| 44 | Pregnant women are able to get all the necessary medicines easily in government owned hospitals |  |  |  |  |  |
| 45 | The medical equipment is adequate in government owned hospitals offering free maternal healthcare services |  |  |  |  |  |
| 46 | The rooms are adequate in government owned hospitals offering free child delivery |  |  |  |  |  |
| 47 | The doctors are adequate in government owned hospitals offering free child delivery |  |  |  |  |  |
|  | **Hygiene and cleanliness of Hospitals** |  |  |  |  |  |
| 48 | The cleanliness of the public hospitals offering free child delivery is adequate |  |  |  |  |  |
| 49 | The conditions of the toilets in public hospitals offering free child delivery are good |  |  |  |  |  |
|  | **Privacy** |  |  |  |  |  |
| 50 | Privacy of pregnant women is ensured in hospitals offering free maternal healthcare services |  |  |  |  |  |
|  | **Accessibility** |  |  |  |  |  |
| 51 | The public hospital offering free child delivery are always open |  |  |  |  |  |
| 52 | Doctors are always available in public hospital offering free child delivery |  |  |  |  |  |
| 53 | Nurses are always available in public hospital offering free child delivery |  |  |  |  |  |
| 54 | The distance to the health centre that offer free child delivery is adequate |  |  |  |  |  |
| 55 | There are adequate means of transportation in case of complications that need for referrals in the closest facility that offers free maternal healthcare services |  |  |  |  |  |

**SECTION 4: BELIEFS ABOUT FREE AND FREE MATERNAL HEALTHCARE**

Please state the extent to which you agree or disagree with the following statements by choosing from Strongly Agree SA, Agree A, Not Sure NS, Disagree D, Strongly Disagree SD.

| 56 | If child delivery is free, it could come back to affect the mother or the baby negatively one-way or the other. |  |  |  |  |  |
| --- | --- | --- | --- | --- | --- | --- |
| 57 | The traditionally belief that a token must always be paid for treatment in order for the disease not to come back is applicable to child delivery |  |  |  |  |  |
| 58 | A token must always be paid for child delivery |  |  |  |  |  |
| 59 | One can get good quality at no cost |  |  |  |  |  |
| 60 | Things that are offered for free always have a hidden cost. |  |  |  |  |  |
| 61 | Free public services are mostly of inferior quality |  |  |  |  |  |
| 62 | One should beware of things that are free! |  |  |  |  |  |
| 63 | Free services are of good quality |  |  |  |  |  |
| 64 | The best things in life are free |  |  |  |  |  |
| 65 | I rather use free things even if it is of inferior quality |  |  |  |  |  |

**SECTION 5: EXPERIENCE OF WOMEN UTILIZING FREE MATERNAL HEALTHCARE**

*This section is only for women that gave birth in a free maternal healthcare facility*

| 66 | Were you attended to early? | Yes  No | 1  2 |
| --- | --- | --- | --- |
| 67 | How would you assess the attitude of nurses? | Very Good  Good  Average  Bad  Very Bad | 1  2  3  4  5 |
| 68 | How would you assess the attitude of the doctors? | Very Good  Good  Average  Bad  Very Bad | 1  2  3  4  5 |
| 69 | How would you assess the cleanliness hospital wards, and theaters? | Very clean  Clean  Moderately clean  Dirty  Very Dirty | 1  2  3  4  5 |
| 70 | What was the condition of the toilets? | Very good  Good  Moderate  Bad  Very bad | 1  2  3  4  5 |
| 71 | Was your privacy ensured? | Yes  No | 1  2 |
| 72 | Are all the medicines required available in the hospital? | Yes  No | 1  2 |
| 73 | How easy were you able to get all the necessary medicine? | Very Easy  Easy  Moderately Easy  Not Easy | 1  2  3  4 |
| 74 | How far is the distance of the closest government hospital to your home? | Very Far  Far  Not far | 1  2  3 |
| 75 | Was the delivery 100 % free | Yes  No | 1  2 |
| 76 | If No, How much were you required to pay | ………………… | 96 |
| 77 | What was the payment for? | Medicines  Caesarean section  Bed  Blood  Other Delivery Items | 1  2  3  4  5 |
| 78 | How affordable is the cost you were charged? | Affordable  Not affordable | 1  2 |
| 79 | Were drugs and delivery kits available for free? | Yes  No | 1  2 |
| 80 | Were you satisfied with the quality of services provided? | Yes  No | 1  2 |
| 81 | If no Why? | …………………………………………………….………………………..….................................... | 99 |

**Awareness and use of family planning methods**

Now I would like to talk about family planning - the various ways or methods that a couple can use to delay or avoid a pregnancy. Have you ever heard of (METHOD)?

Write the code in the space

| 1 | Male Sterilization (Y/N)  IUD (Y/N)  Injectables (Y/N)  Implants (Y/N)  Pill (Y/N)  Condom (Y/N)  Female Condom (Y/N)  Emergency Contraception (Y/N)  Standard Days Method (Y/N)  Lactational Amenorrhea Method (Y/N)  Rhythm Method (Y/N)  Withdrawal (Y/N)  Other Modern Method (Y/N)  Other Traditional Method (Y/N) |  |  |
| --- | --- | --- | --- |
| 2 | Have you heard of any other ways or methods that women or men can use to avoid pregnancy? If yes mention it | Write the response here  ……………………………….. | 98 |
| 3 | Have you ever do something or using any method to delay or avoid getting pregnant? | Yes  No | 1  2 |
| 4 | Are you or your partner currently doing something or using any method to delay or avoid getting pregnant? | Yes  No | 1  2 |
| 5 | Which method are you using? Probe | Female Sterilization Y/N  Male Sterilization Y/N  IUD Y/N  Injectables Y/N  Implants Y/N  Pill Y/N  Condom Y/N  Female Condom Y/N  Emergency Contraception Y/N  Standard Days Method Y/N  Lactational Amenorrhea Method Y/N  Rhythm Method Y/N  Withdrawal Y/N  Other Modern Method Y/N  Other Traditional Method Y/N | 1  2  3  4  5  6  7  8  9  10  11  12  13  14  14  15 |
| 6 | If not using any method probe for reason for use | Lack of knowledge  Can not afford it  It could be harmful to the body  It is not effective  Currently pregnant  Others specify ----------- | 1  2  3  4  5  6  96 |

**Contraceptive discontinuation**

I would like to ask you some questions about the times you or your partner may have used a method to avoid getting pregnant during the last few years.

Fill in the responses

| 2 | When did you start using that method? How long after the birth of (NAME)? |  |  |  |
| --- | --- | --- | --- | --- |
| 3 | How long did you use the method then? |  |  |  |
| 4 | Why did you stop using the (METHOD)? |  |  |  |
| 5 | Did you become pregnant while using (METHOD), or did you stop to get pregnant, or did you stop for some other reason? |  |  |  |

| 1 | Female Sterilization. PROBE: Women can have an operation to avoid having any more children. | Yes  No | 1  2 |
| --- | --- | --- | --- |
| 2 | Male Sterilization. PROBE: Men can have an operation to avoid having any more children. | Yes  No |  |
| 3 | IUD. PROBE: Women can have a loop or coil placed inside them by a doctor or a nurse which can prevent pregnancy for one or more years. | Yes  No |  |
| 4 | Injectables. PROBE: Women can have an injection by a health provider that stops them from becoming pregnant for one or more months. | Yes  No |  |
| 5 | Implants. PROBE: Women can have one or more small rods placed in their upper arm by a doctor or nurse which can prevent pregnancy for one or more years. | Yes  No |  |
| 6 | Pill. PROBE: Women can take a pill every day to avoid becoming pregnant. | Yes  No |  |
| 7 | Condom. PROBE: Men can put a rubber sheath on their penis before sexual intercourse. | Yes  No |  |
| 8 | Female Condom. PROBE: Women can place a sheath in their vagina before sexual intercourse. | Yes  No |  |
| 9 | Female Condom. PROBE: Women can place a sheath in their vagina before sexual intercourse. | Yes  No |  |
| 10 | Emergency Contraception. PROBE: As an emergency measure, within three days after they have unprotected sexual intercourse, women can take special pills to prevent pregnancy. | Yes  No |  |
| 11 | Rhythm Method. PROBE: To avoid pregnancy, women do not have sexual intercourse on the days of the month they think they can get pregnant. | Yes  No |  |
| 12 | Withdrawal. PROBE: Men can be careful and pull out before climax. | Yes  No |  |
| 13 | Lactational Amenorrhea Method (LAM). PROBE: Up to six months after childbirth, before the menstrual period has returned, women use a method requiring frequent breastfeeding day and night | Yes  No |  |
| 14 | Have you heard of any other ways or methods that women or men can use to avoid pregnancy? | Yes  No |  |
| 15 | Are you or your partner currently doing something or using any method to delay or avoid getting pregnant? | Yes  No |  |
| 16 | Which method are you using? | Female Sterilization Y/N  Male Sterilization Y/N  IUD Y/N  Injectables Y/N  Implants Y/N  Pill Y/N  Condom Y/N  Female Condom Y/N  Emergency Contraception Y/N  Standard Days Method Y/N  Lactational Amenorrhea Method Y/N  Rhythm Method Y/N  Withdrawal Y/N  Other Modern Method Y/N  Other Traditional Method Y/N | 1  2  3  4  5  6  7  8  9  10  11  12  13  14  14  15 |
| 17 | If not using any method probe for reason | …. |  |
