# Supplementary material for: Determinants of condom use among parous women in North Central and South Western Nigeria: a cross-sectional survey
Source: BMC Res Notes. 2018 Jul 13;11:467. doi: 10.1186/s13104-018-3573-5 (PMC6044001; doi:10.1186/s13104-018-3573-5)
Supplement: Supplementary file 2 — Additional file 2: Figure S1. Condom use prevalence. [file 13104_2018_3573_MOESM2_ESM.docx]

* FP means Family planning

Figure S1. Condom use prevalence
